# A novel, dual-contrast in-vivo MR imaging method with principal component analysis reliably quantifies lipid-rich necrotic core and collagen in human carotid atherosclerotic plaques

**DOI:** 10.1186/1532-429X-11-S1-O45

**Published:** 2009-01-28

**Authors:** Zhen Qian, Sarah Rinehart, Laura J Murrieta, Gustavo Vasquez, Patrick M Battey, Szilard Voros

**Affiliations:** Piedmont Heart Institute, Atlanta, GA USA

**Keywords:** Principal Component Analysis Analysis, Plaque Composition, Human Carotid, Feridex, Imaging Pulse Sequence

## Background

In-vivo multi-spectral imaging of human carotid plaques using different pulse sequences, with and without exogenous contrast agents, has been implemented. However, an in-vivo dual-contrast approach with small paramagnetic iron oxide (SPIO) and gadolinium at multiple timepoints with principal component analysis (PCA) has not been previously performed.

## Purpose

To develop a novel, PCA-based method for the detection of lipid-rich necrotic core (LRNC) and collagen in human carotid plaques utilizing a dual-contrast approach. We hypothesized that LRNC and collagen can be reliably identified based on different signal characteristics with different exogenous contrast agents and multiple different imaging pulse sequences.

## Methods

10 pts scheduled for carotid endarterectomy (CEA) were imaged at 1.5 T with a dedicated small surface coil. T1, T2 and inversion recovery delayed hyperenhancement (DHE) images with magnitude/phase reconstruction were obtained before contrast, immediately and 24 hours after 0.05 cc/kg of SPIO (Feridex) and after 30 cc of Gd. Imaging parameters were: T1; TR: 1500 ms, TE 10 ms, slice 3 mm, matrix 320 × 320, averages 3; T2; TR: 2500 ms, TE 92 ms, slice 3 mm, matrix 320 × 320, averages 4; DHE; TR: 745 ms, TE 3.5 ms, slice 6 mm, matrix 192 × 192, averages 1. Corresponding histological sections from CEA specimens were stained with Movat's pentachrome for identification of LRNC and collagen.

For a training dataset, ROI in two pts were normalized to foreground median intensity and histopathological specimens were non-rigidly registered to the MR images using anatomical landmarks and a thin-plate spline-based image morphing algorithm (Panel C). Plaque composition masks consisting of 6 classes (LRNC [red], calcium [yellow], fibrous collagen [green], proteoglycans [light blue], elastin [grey], and fibrin [purple]) were created based on the registered histological images (Panel D). All 4 sequences at all 4 timepoints were independently tested for the identification of plaque composition. A more comprehensive PCA analysis utilizing all pulse sequences at all timepoints was also performed. Signal intensity (SI) statistics are expressed as mean ± SD. The performance of composition identification was measured by comparing the predicted compositions with the 2-class mask, and was evaluated using the two-tailed t-test p value, and the area under the ROC curve.

## Results

**Figure 1:**
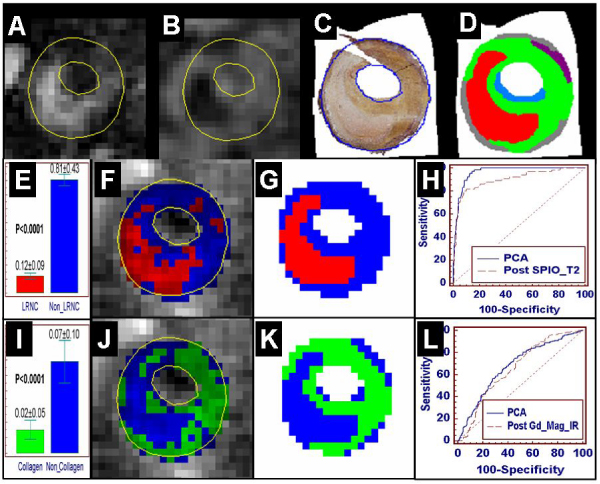
Figure 1

See Figure 1. SI was significantly higher in LRNC on T2 images immediately after SPIO (Panel A) compared to other tissues (56.66 ± 18.52 vs. 26.21 ± 12.74, p < 0.0001). PCA showed significant difference between LRNC and non-LRNC tissue (p < 0.0001) (Panel E). The percentage of correctly identified pixels was 80.4%. Predicted total area for LRNC was 71 pixels (22.22 mm^2^), compared to 66 pixels (20.65 mm^2^) on the manually identified mask (Panel F, G). ROC curve analysis showed that PCA (AUC: 0.96) was significantly better than the single best approach (T2 immediately post-SPIO; AUC: 0.90) (p = 0.016) (Panel H).

SI was significantly higher in collagen on the post-Gd DHE magnitude images (Panel B) (36.56 ± 8.97 vs. 30.21 ± 12.80, p < 0.0001). PCA showed significant difference between collagen and non-collagen tissue (p < 0.0001) (Panel I). The percentage of correctly identified pixels was 65.8%. Predicted total area for collagen was 104 pixels (32.54 mm^2^), compared to 98 pixels (30.67 mm^2^) on the manually identified mask (Panel J, K). ROC curve analysis showed that PCA (AUC: 0.68) was similar to the single best approach (Post-Gd magnitude DHE; AUC: 0.66) (p = 0.562) (Panel L).

## Conclusion

LRNC and collagen can be reliably identified in-vivo in human carotid atherosclerotic plaques using our novel, dual-contrast approach; a different pulse sequence with different exogenous contrast is optimal for the identification of different tissue components. A more sophisticated PCA analysis is significantly better than the evaluation of a single pulse sequence at a single timepoint for the identification of LRNC. Such sophisticated plaque analysis can now be applied to clinical outcomes studies and for the evaluation of the effects of pharmaceutical agents for the modification of atherosclerosis.

